# Ultrasound and magnetic resonance imaging as diagnostic tools for sarcopenia in immune-mediated rheumatic diseases (IMRDs)

**DOI:** 10.1007/s11547-022-01560-y

**Published:** 2022-09-20

**Authors:** Fausto Salaffi, Marina Carotti, Andrea Di Matteo, Luca Ceccarelli, Sonia Farah, Catalina Villota-Eraso, Marco Di Carlo, Andrea Giovagnoni

**Affiliations:** 1 Rheumatology Clinic, Università Politecnica Delle Marche, Jesi (Ancona), Italy; 2 Department of Radiological Sciences, Radiology Clinic, Azienda Ospedaliera Universitaria, Ospedali Riuniti di Ancona, Ancona, Italy; 3 Department of Interventional and Diagnostic Radiology, Azienda Ospedaliero-Universitaria Sant’Anna, Ferrara, Italy; 4 Department of Rheumatology, Universidad de La Sabana, Chia, Colombia

**Keywords:** Sarcopenia, Ultrasound, Magnetic resonance imaging, Immune-mediated rheumatic diseases

## Abstract

Sarcopenia is characterized by loss of muscle mass, altered muscle composition, fat and fibrous tissue infiltration, and abnormal innervation, especially in older individuals with immune-mediated rheumatic diseases (IMRDs). Several techniques for measuring muscle mass, strength, and performance have emerged in recent decades. The portable dynamometer and gait speed represent the most frequently used tools for the evaluation of muscle strength and physical efficiency, respectively. Aside from dual-energy, X-ray, absorptiometry, and bioelectrical impedance analysis, ultrasound (US) and magnetic resonance imaging (MRI) techniques appear to have a potential role in evaluating muscle mass and composition. US and MRI have been shown to accurately identify sarcopenic biomarkers such as inflammation (edema), fatty infiltration (myosteatosis), alterations in muscle fibers, and muscular atrophy in patients with IMRDs. US is a low-cost, easy-to-use, and safe imaging method for assessing muscle mass, quality, architecture, and biomechanical function. This review summarizes the evidence for using US and MRI to assess sarcopenia.

## Introduction

Muscle mass and function decline rapidly in sarcopenic subjects [1, 2]. Sarcopenia affects the elderly, but not solely [3]. In immune-mediated rheumatic diseases (IMRDs) such as rheumatoid arthritis (RA), psoriatic arthritis (PsA), ankylosing spondylitis (AS), systemic lupus erythematosus (SLE), systemic sclerosis (SSc), vasculitides, and in aging disorders, sarcopenia has recently been added to the International Classification of Diseases (ICD-10) as a comorbidity [4]. As a result of mobility problems, sarcopenia can lead to decreased quality of life, loss of independence or the need for long-term care [5–12]. It has a higher mortality rate (pooled odds ratio of 3.6) than the general population, according to a recent study [13]. Sarcopenia also has a financial cost [14], increasing the risk of hospitalization and the expense of care [15]. Sarcopenic individuals are five times more likely than non-sarcopenic patients to have higher hospital charges [16]. In 2000, the projected direct health care cost of sarcopenia in the USA was $18.5 billion ($10.8 billion for males, $7.7 billion for women), accounting for around 1.5% of overall health care spending. According to a sensitivity study, the expenses might range from $11.8 billion to $26.2 billion. Each sarcopenic male spent $860 on health care and each sarcopenic woman spent $933 on health care. A 10% decrease in sarcopenia prevalence would result in annual health care cost savings of $1.1 billion (dollars adjusted to 2000 rate) in the USA [17].

### Epidemiology of sarcopenia in older community-dwelling and IMRDs

Sarcopenia vastly outnumber frailty in the general population. After age 50, muscle mass diminishes by 1–2% per year. Muscle strength falls by 1.5% between 50 and 60, and by 3% thereafter [18]. Sarcopenia prevalence varies depending on the population examined and the classification criteria utilized [19, 20], regarding 5–13% of adults aged 60–70. The ratio rises to 11–50% for those above 80 [18]. Sarcopenia affects around 50 million people globally and is anticipated to reach 200 million in the next 40 years [21]. According to the European Working Group on Sarcopenia in Older People (EWGSOP2) definition and standards [3, 23], sarcopenia affects 4.6% of male group housing residents aged 68–76 in the UK [22]. In a cross-sectional observational research of 730 elderly people, those with chronic conditions (endocrine disorders, cancers, heart failure, cognitive impairment, Parkinson's disease, renal failure, peripheral artery disease, and hip fracture) had greater rates of sarcopenia [24].

IMRDs might represent risk factors for sarcopenia [25]. Pro-inflammatory cytokines including interleukin-6 and tumor necrosis factor (TNF) promote systemic inflammation, which leads to sarcopenia [26]. Sarcopenia (about 20%) and pre-sarcopenia were studied in Italian RA, PsA, and AS patients. Although sarcopenia was seen in all three disorders, pre-sarcopenia was shown to be more common in PsA and AS (25.7%) than in RA [27]. Sarcopenia is more common in Asian RA patients (37.1%) [28], and in North African AS patients (34.3%) [29]. Other cross-sectional studies indicated that RA patients had considerably more sarcopenia than controls [30–35]. Sarcopenia was found in RA patients in these studies in a range of 10–45%, with a median of 29%. A recent comprehensive literature review and meta-regression analysis of 3.140 RA patients revealed 31% sarcopenia [36].

Patients with AS had pre-sarcopenia, sarcopenia (as defined by the EWGSOP), and cachexia [29]. Higher disease activity (BASDAI) and lower bone mineral density (BMD) were associated with sarcopenia and cachexia. A cross-sectional study indicated that 20% of people with spondyloarthritis had sarcopenia. This research comprised 22 AS patients and 70 PsA patients from 40 to 75 years old. Sarcopenia was found in 22.7% of AS and 20.0% of PsA patients utilizing the skeletal muscle mass index (SMI) and handgrip force [27]. According to the EWGSOP, 34.3% of Moroccan AS patients and 62% of Portuguese AS patients had sarcopenia. SMI correlated negatively with BASDAI and Bath Ankylosing Spondylitis Function Index (BASFI) [37]. The frequency of sarcopenia in postmenopausal female PsA patients utilizing the SMI is 40–50% [38, 39].

Sarcopenia is seen in 17.4% of SLE patients [31]. Three studies looked at SSc sarcopenia prevalence. The prevalence of sarcopenia was 20.7% using the SMI [40], and 22.5% using the EWGSOP criteria [41]. Another study revealed prevalence rates of 41.9 and 54.8% using SMI and handgrip strength criteria [42].

### Definition and diagnosis of sarcopenia

Sarcopenia has several definitions [3–5, 21, 43, 44], but no consensus has been reached. Sarcopenia is “a condition of progressive and generalized loss of skeletal muscle mass and strength with a risk of adverse consequences such as physical weakness, poor quality of life, and death” according to the EWGSOP [21]. Low muscle mass alone indicates pre-sarcopenia, loss of muscular strength or performance suggests sarcopenia, and the combination of all three characteristics indicates extreme sarcopenia (Table 1).

**Table 1:** EWGSOP operational definition of sarcopenia (adapted from [21])

*Criteria*
1. Low muscle strength
2. Low muscle quantity or quality
3. Low physical performance
*Interpretation*
Probable sarcopenia is identified by the presence of Criterion 1
Diagnosis is confirmed by additional documentation of Criterion 2
If Criteria 1, 2 and 3 are all met, sarcopenia is considered severe

**Fig. 1:**
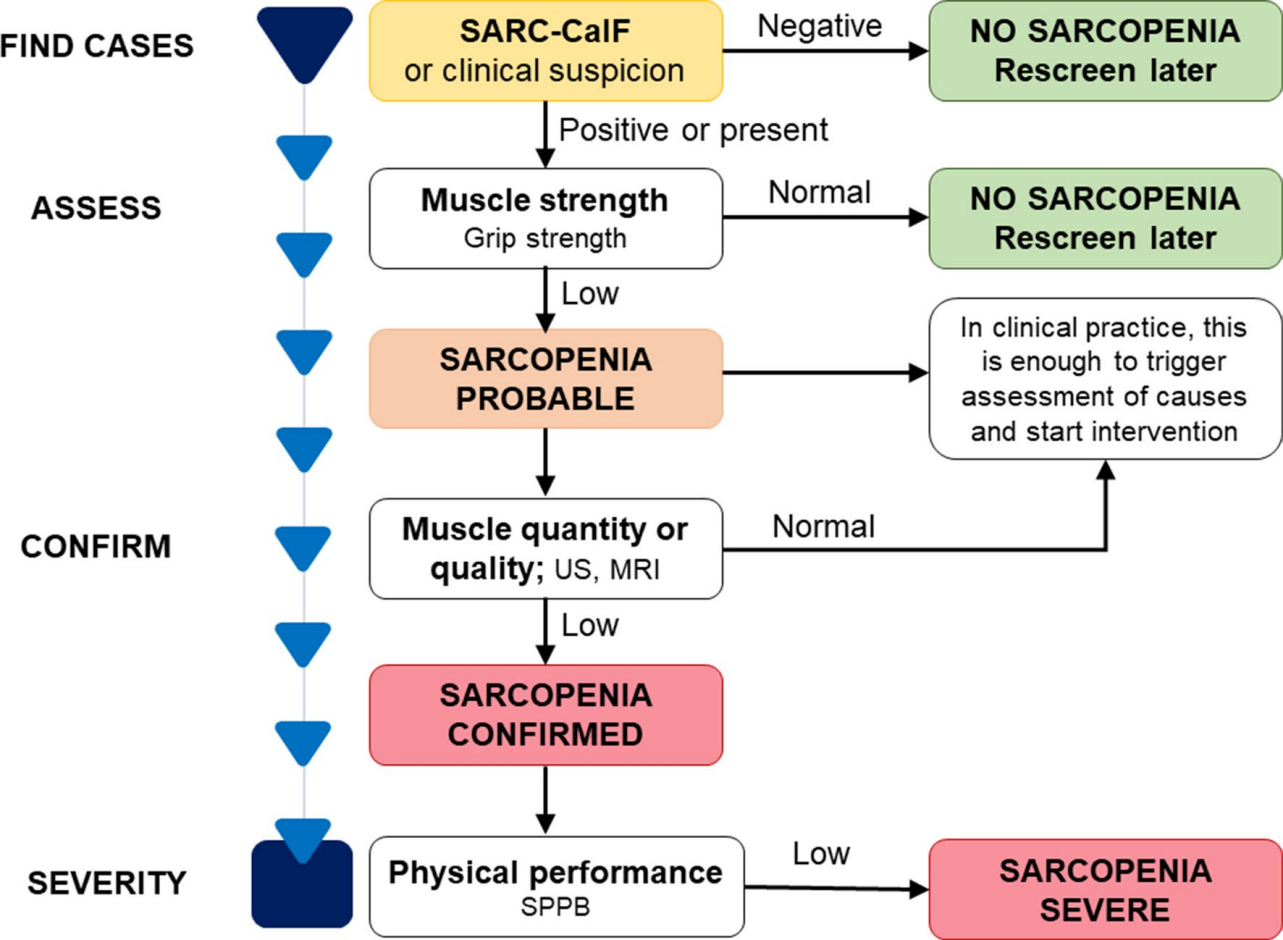
Sarcopenia assessment flowchart for case-finding, making a diagnosis and quantifying severity in practice (adapted from [3]). Abbreviations: SARC-CalF = Strength, Assistance with walking, Rise from a chair, Climb stairs, Fall and Calf Circumference; SPPB = Short Physical Performance Battery; US = Ultrasound; MRI = Magnetic Resonance Imaging

The Working Group reconvened in 2018 (EWGSOP2) to modify the initial criteria to incorporate ten years of scientific and clinical research. The amended EWGSOP recommends measuring muscle mass, strength, and quality [3]. In particular, EWGSOP2 recognized poor physical performance as an indication of severe sarcopenia and utilized low muscle quantity and quality to corroborate the diagnosis. EWGSOP2 also modified the clinical methodology for sarcopenia identification, diagnosis, and severity assessment. The updated EWGSOP2 guidelines seek to educate the public about sarcopenia and its consequences. This change was made to align the algorithm with the 2018 sarcopenia concept and make it easier to use in clinical settings. Figure 1 shows a modified version of the EWGSOP2 flowchart, and it could be proposed for the identification of sarcopenia in patients with IMRDs.

The Strength, Assistance with walking, Rise from a chair, Climb stairs and Falls (SARC-F) questionnaire is recommended by EWGSOP2 for sarcopenia patients to self-report. SARC-F looks to be appropriate for community health care. The SARC-F is a 5-item questionnaire used to determine sarcopenia risk [45]. They rate their strength, walking abilities, standing up from a chair and stair climbing, as well as falls. In clinical groups with sarcopenia suspicion, clinicians may use a more systematic case-finding instrument, such as the Strength, Assistance with walking, Rise from a chair, Climb stairs, Fall and Calf Circumference (SARC-CalF), which incorporates calf circumference (CC) [46, 47]. Low handgrip strength (HGs) is currently the most reliable indicator of muscular function in EWGSOP2’s 2018 definition. Sarcopenia is characterized by HGs loss [48–50]. HGs have been linked to disease activity, joint injury, disability, and functional impairments in IRMDs and fibromyalgia [51, 52]. HGs may assess a patient’s ability to return to work, measure progress, and compare the efficacy of different treatment options [53]. Moreover, the French Very Early Rheumatoid Arthritis study indicated that decreasing HGs is linked to a higher economic burden in individuals with RA [54].

To increase the sensitivity and specificity of current diagnostic criteria, studies should be conducted using standardized and reliable imaging methods [55]. Muscle mass analysis and measuring methodologies may provide a variety of results. Depending on the system used, total lean mass (body weight minus body fat), appendicular lean muscle mass (aLM), or both can be measured using bioelectrical impedance analysis (BIA), computed tomography (CT), ultrasound (US), magnetic resonance imaging (MRI), and dual-energy X-ray absorptiometry (DXA) [21, 55–57]. In terms of reliability, radiation exposure, amount of time to complete the examination and analyze the data, availability and complexity of the equipment required, costs, and applications, these approaches vary significantly.

The EWGSOP2 recommends the use of DXA as a tool to diagnose sarcopenia in clinical practice. Although DXA has same advantages, such as the relatively low radiation exposure and it is more cheater compared to CT scan, it has several limitations. DXA has proven to have low accuracy in the presence of edema and altered hydration status, in estimating truncal fat and muscle due to the inability to separate intra-abdominal organs and in evaluating the extent of sarcopenia or the presence of obesity from the amount of fat and muscle extrapolated from arms and legs.

### Imaging methods for the diagnosis of sarcopenia

In a community-based vulnerable older adult population, sarcopenia seems to be best diagnosed with US and MRI (Table 2) [55–59]. The mobility and absence of ionizing radiation exposure of US separates it from other techniques. MRI is often used to quantify skeletal muscle efficiency, especially intramyocellular lipid. Both approaches can assess muscle thickness, intramuscular fat infiltration, and other biochemical indices of muscle quality since muscle and fat are clearly distinguished [56, 58]. Variations in echo intensity (EI) are associated with increased intramuscular fiber and fat tissue [59–64]. Researchers who employed computer-aided gray scale analysis to determine muscle quality say the EI increases intramuscular adipose and fibrous tissue. In recent years, pixel/voxel threshold distinctions between muscle and other tissues have been established semiautomatically [60–64]. In addition to frailty, quantitative MRI data may identify variations in muscle function across age groups [65–69]. Using automated subcutaneous fat and muscle segmentation, multiparametric MRI has recently shown promise in measuring subcutaneous adipose tissue (SAT) and intermuscular adipose tissue (IMAT) [70]. The latter approach cannot reliably measure intramyocellular lipids [71]. These two methodologies demonstrate how sarcopenia imaging has evolved from basic anatomical or structural measurement to a new level that permits functional dissection of muscle tissue.

**Table 2:** Advantages and limitations of the two different modalities (MRI and US), used in estimation of skeletal muscle mass (adapted from [55])

Technique	Advantages	Limitations
MRI	No radiation exposure	High equipment costs
	Good for imaging soft tissues	Time consuming
	Able to review images after scanning	Limited accessibility for frail community-based patients and those with cognitive impairment
	Thorough image acquisition	Confined space in scanner
	Body mass composition differentiation	Low availability
		No definite low muscle mass thresholds
	High spatial resolution	Cannot use if patient has metal work/some pacemakers
	Accuracy	
	Suitable for long-term follow-up, progression monitoring	Requires interpretation by radiologist
	Capable of detecting changes in muscle structure	Lack of standardized assessment protocol
	Cross-sectional imaging	Longer image acquisition time, complex post-processing
	Muscle edema and myosteatosis detection	Lack of portability
		Complex post-processing
		Controindications
US	Extremely safe	Variety of probes required to achieve varying depth/resolution
	Low cost	
	No radiation exposure	Limited use in obese patients
	Ability to perform dynamic testing	Operator skills required
	Portable	Low reproducibility
		Low accuracy
	Cost-effective	No criteria for diagnosis of low muscle mass
		Fixed anatomical landmarks needed
	Quick to perform (short image acquisition time)	Correlation with functional parameters still unclear
	Suitable in all patient groups	Results depending on the type of software used to interpret images
	Can be interpreted at bedside by a lay sonographer (real-time imaging)	Studies focused on elderly subjects are lacking

### The role of ultrasound as a diagnostic tool for sarcopenia

This narrative review discusses several studies that have explored the value of US in identifying non-myositis-related muscle involvement in patients with IMRDs, assessing the potential and limits of US in screening and diagnosing sarcopenia in individuals with these disorders.

**Fig. 2:**
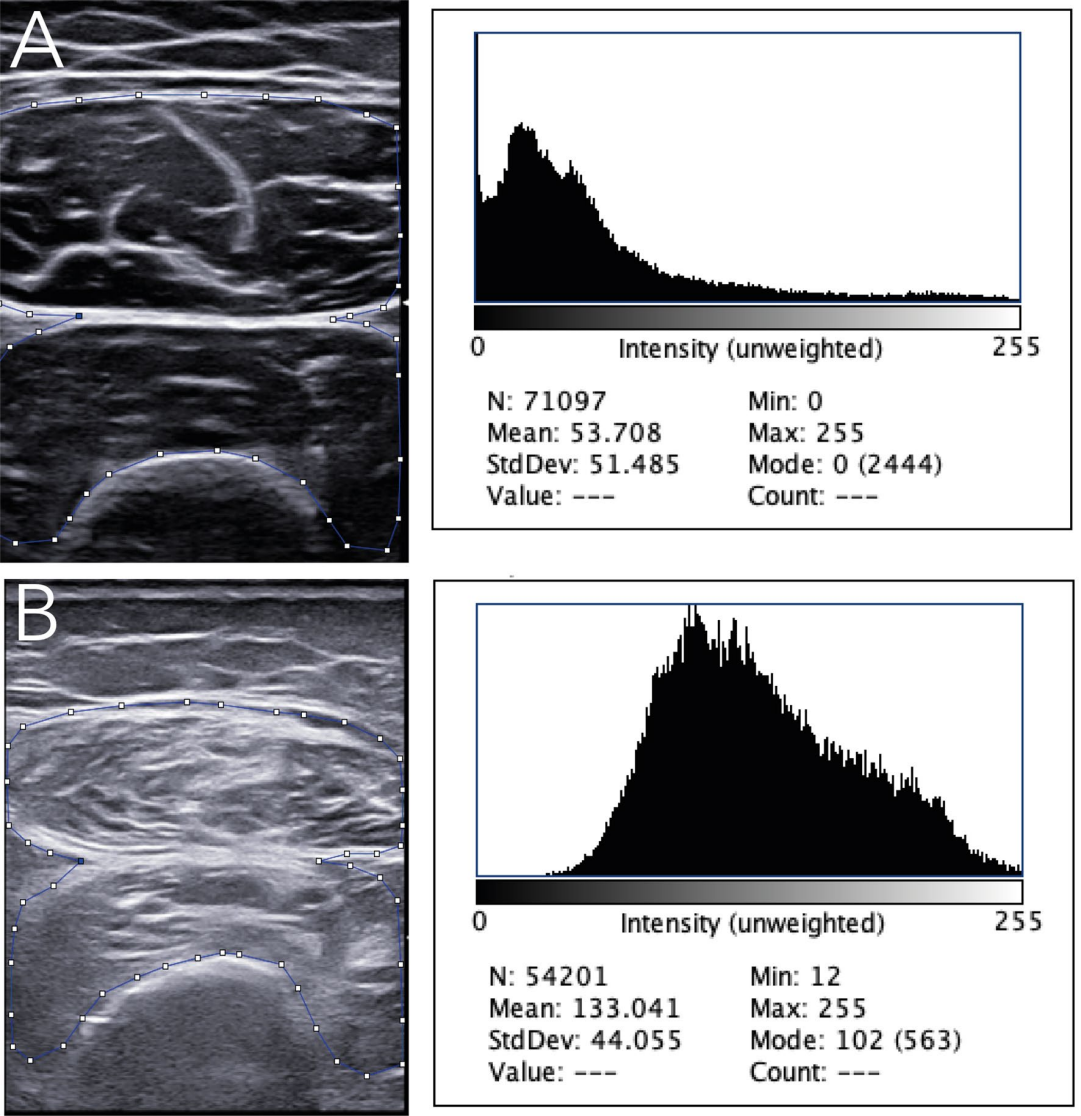
Muscle echogenicity in grayscale and histographic analysis in a healthy subject (**a**) compared to a patient with systemic sclerosis (**b**). An ultrasound transverse scan image of the rectus femoris (rf) and vastus intermedius (vi) showing increased muscle echogenicity in the patient with systemic sclerosis in comparison with the healthy subject (i.e., grade III of the Heckmatt scale, marked increased muscle echo with reduced bone echo vs grade I of the Heckmatt scale, normal hypoechoic muscle) [76]. The small squares and lines indicate the region of interest for grayscale image analysis with histograms

The potential usefulness of US in the diagnostic work-up of sarcopenia mainly relies on the capacity of this imaging technology to examine numerous aspects of muscle changes. US has proven the potential to detect qualitative (i.e., muscle echogenicity indicative of muscle fibrosis or fatty replacement), and biomechanical muscle changes (i.e., pennation angle and fascicle length) in patients with (or ‘at-risk’ of) sarcopenia, mainly elderly patients and patients with neuromuscular disorders, but also patients with IMRDs [72–74]. Several approaches have been developed to measure US muscle echogenicity [75]. The most widely chosen is the visual approach, which is a subjective and intuitive appraisal of muscle echogenicity in relation to the surrounding tissues, such as the subcutaneous tissue. One of most is represented by the Heckmatt score, a 4-grade semiquantitative measure which was created in pediatric patients with neuromuscular illnesses in 1982 [76]. Quantitative metrics of echogenicity on US images, such as histographic analysis, may also be utilized. This sort of technique is based on software that estimates the number of pixels on grayscale images (Fig. 2). Shear-wave elastography (SWE) is a relatively recent US technique that analyzes muscle physiological parameters by giving a quantitative measure of muscle elasticity [77, 78].

The majority of the studies on US were carried out in patients with RA [79–85], while only a very few have investigated US muscle involvement in patients with connective tissue diseases [86, 87].

In a study by Matschke and colleagues, 14 cachectic RA patients (defined as reduced appendicular lean mass by whole-body dual-energy X-ray absorptiometry) were evaluated for US parameters (vastus lateralis fascicle length, pennation angle, and cross-sectional area [CSA]). Physical functions (i.e., sit-to-stand, foot-up-and-go, 50-foot walk, and single-leg balance) were impaired in RA patients compared to healthy controls. The muscle-specific force and activation capacity of the two groups did not vary significantly. It is possible that cachectic RA patients with poor physical performance, reduced muscle mass, and US muscle abnormalities nonetheless have these physiological features. Sadly, the authors did not study (or publish) the link between US results, physical function, and muscular mass.

Another research compared 35 RA patients with 35 age- and sex-matched healthy controls, in terms of athletic performance, US vastus lateralis muscle strength, and fascicle length. Less muscle thickness (23.3%) and pennation angle (14.1%) were seen in RA patients as compared to healthy controls in the vastus lateralis muscle, but no variations were found in fascicle length between the two groups. Less physical function (TUG test) and knee-extensor muscle strength were found in RA patients compared to healthy controls. Neither the US findings nor the clinical characteristics of RA patients (HAQ, DAS-28 joints, glucocorticoid treatment, disease duration, and VAS pain) were associated with each other.

Similarly, another research looked at the relationship between quadriceps muscle US morphology (muscle thickness and pennation angle) and clinical characteristics, muscular strength (grip strength test) and physical function (HAQ, TUG test) in 55 women with RA [82]. Unlike the previous trial, this research supported the use of US in detecting RA patients with impaired muscular strength and physical function. The loss of quadriceps US muscle thickness was linked with age, illness duration, and hand grip strength (negative association). Also, decreased vastus intermedius US muscle thickness and reduced rectus femoris pennation angle correlated with lower DAS-28 scores. Finally, a decreased rectus femoris US muscle thickness was associated with a prolonged chair stand test, as was a decreased vastus lateralis pennation angle. The size of the population may be one factor for the disparity in outcomes between these two investigations.

Tada and colleagues have studied the function of muscle US in detecting sarcopenia and obesity in RA patients [83]. These researchers investigated the relationship between sarcopenia (as defined by the Asian Working Group for Sarcopenia—AWGS) and obesity (as measured by a bioelectrical impedance analyzer) in 84 individuals with RA. The AWGS reported a 22.6% prevalence of sarcopenia in this investigation. In addition, the authors identified US cutoffs of reduced muscle mass (24.7 mm in men and 19.7 mm in women) that had 52.6% sensitivity, specificity, positive and negative predictive values for sarcopenia diagnosis, respectively. Obesity was seen in 28.6% of RA patients. Obesity and US fat thickness correlated significantly (men r = 0.66, women r = 0.62, *p* < 0.001). The authors also determined US fat thickness cutoffs (8.1 mm for males and 14.6 mm for women) with 96.7% sensitivity, specificity, and positive and negative predictive values for obesity diagnosis. The findings of this research suggest that US might be used to screen for sarcopenia and obesity in RA patients, with implications for early diagnosis and therapy (e.g., referral to dedicated activity programs, diet).

Yoshida and colleagues recently examined quantitative (CSA) and qualitative (muscle echogenicity) US muscle findings in RA patients with (n = 34) and without (n = 44) sarcopenia (AWGS consensus) with a healthy control group (n = 15) [84]. Physical tests, such as walking speed and the chair stand test, were performed in such populations while body composition was assessed by bioimpedance analysis. Muscle echogenicity and CSA were measured in the biceps brachii, vastus lateralis, and rectus femoris. Muscle echogenicity and CSA were higher in sarcopenic RA patients than in non-sarcopenic RA patients and healthy controls. Furthermore, US CSA and muscle echogenicity correlated with gait speed and skeletal muscle index (i.e., body composition). The combined evaluation of muscle mass (CSA) and muscle echogenicity produced the greatest diagnostic results for sarcopenia, outperforming the single US findings, indicating the value of doing a ‘multimodal' US muscle assessment in RA patients.

Most studies assessing US muscle changes in RA patients focused on quantitative (muscle mass), architectural, and/or biomechanical changes. To our knowledge, just one research looked at muscular stiffness in this group. Alfuraih and colleagues studied SWE muscle stiffness in three RA patient groups (29 newly diagnosed, 33 in clinical remission, and 18 with current illness) and compared the results to a healthy control group [85]. The authors also looked at the relationship between SWE muscle stiffness and participants’ strength and performance. Although RA patients had worse muscular strength and physical performance than healthy people, the difference was not statistically significant, particularly in those with active disease. SWE muscle stiffness has no association with RA disease activity or muscle function. Thus, the authors’ initial hypothesis of changed muscle stiffness in RA and its probable link with disease duration and activity was not supported.

Few studies address US in sarcopenia diagnosis in connective tissue disorders. Sari and colleagues assessed the agreement and association between muscle US and BIA in 93 SSc patients [86]. The authors used US to evaluate the gastrocnemius medialis, rectus femoris, rectus abdominis, external, internal, and transverse abdominis muscles. All muscles except the rectus femoris (r = 0.196; *p* = 0.061) showed a significant connection between decreased US muscle thickness and low muscle mass. A receiver operating characteristic study also established cutoff values for gastrocnemius medialis and rectus abdominis muscle thickness (sensitivity: 92.3% for both; negative predictive value: 97.9% and 97.6%). Finally, decreasing US muscle thickness in all muscles studied was associated with lower grip strength.

Kaya and colleagues compared muscular strength and US muscle architecture (muscle thickness, pennation angle, and fascicle length) in 31 SLE patients to 31 age- and sex-matched healthy volunteers [87]. Interestingly, whereas muscular strength (as measured by isokinetic knee flexion and extension) was decreased in SLE patients compared to healthy controls, US results at the gastrocnemius muscle were not. The vastus lateralis muscles of SLE patients had increased thickness, pennation angle, and fascicle length compared to healthy controls. The authors provided no data on the relationship between US results and muscular strength.

Di Matteo et al. [78] recently investigated muscle mass, quality, and stiffness in SLE patients and healthy controls in the US. The quadriceps muscle thickness was not different between SLE patients and healthy controls (35.2 mm SD 6.8 vs 34.8 mm SD 6.0, *p* = 0.79). Muscle echogenicity was increased in SLE patients (1.7 SD 1.0 vs 0.3 SD 0.5, *p* < 0.01; grayscale analysis with histograms: 87.4 SD 18.8 vs 70.1 SD 14.0, *p* < 0.01). Similarly, SWE was significantly lower in SLE patients compared with healthy subjects [1.5 m/s (IQR 0.3) vs 1.6 m/s (IQR 0.2), respectively, *p* = 0.01).

In recent years, a rising number of studies have shown that US can identify muscle involvement in individuals with IMRDs, potentially affecting early sarcopenia diagnosis and therapy (e.g., referral to dedicated physical activity programs). The apparent variation in the included studies’ approach (e.g., heterogeneity in US procedures, kinds of muscles examined, use of various gold standards) warrants additional examination. Additional efforts are required to design a reliable and cost-effective US technique that can test and analyze all possible elements (qualitative, quantitative, and biomechanical) of muscle participation.

### Diagnostic value of magnetic resonance imaging for sarcopenia

**Fig. 3:**
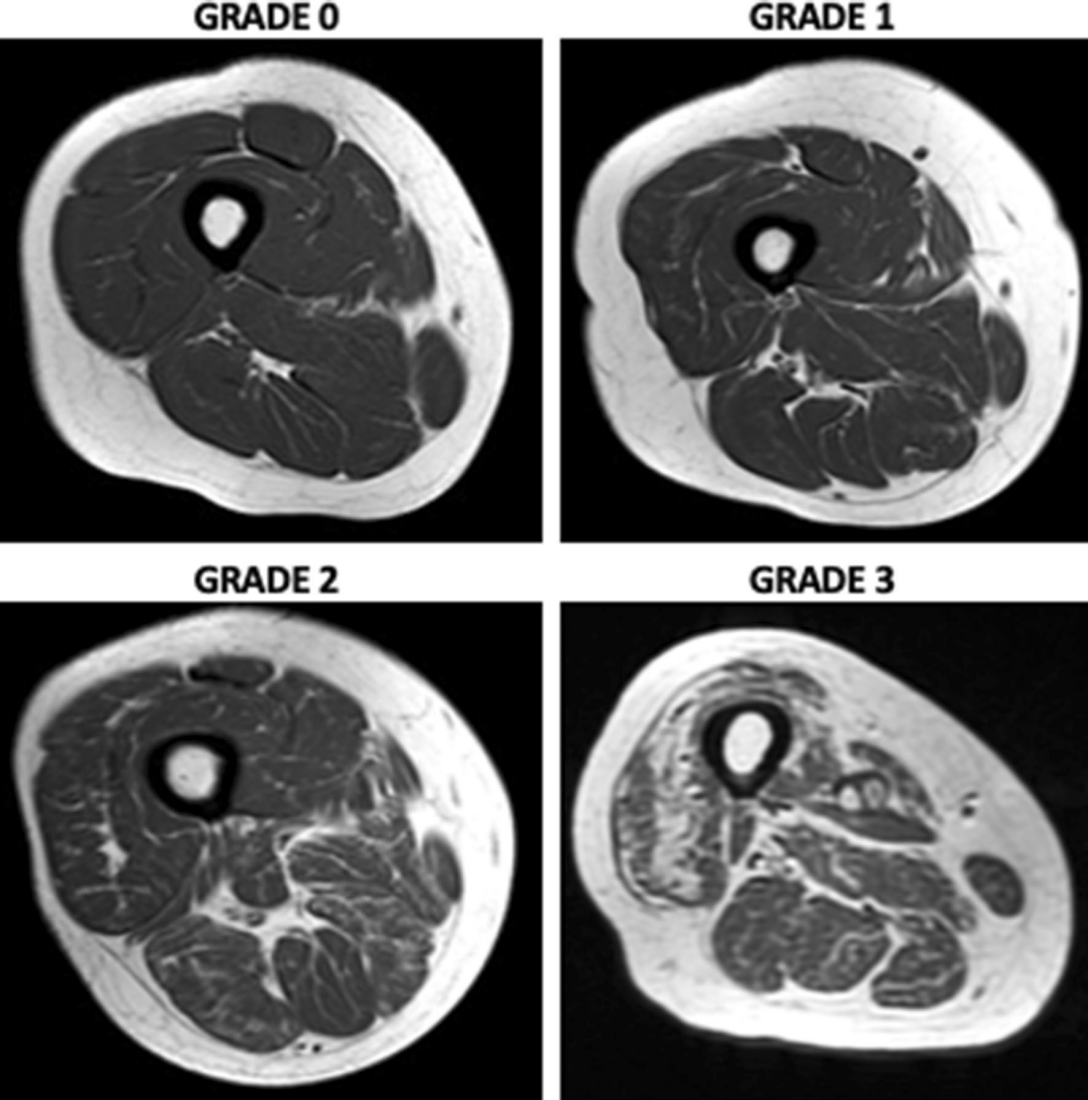
The modified Goutallier classification [93] of fatty infiltration on MRI on a 4-point scale: 0 = normal; completely normal muscle, without any fatty streak; 1 = mild; muscle contains some fatty streaks; 2 = moderate; fatty infiltration is important, but there is more muscle than fat; 3 = severe; more fat than muscle is present

**Fig. 4:**
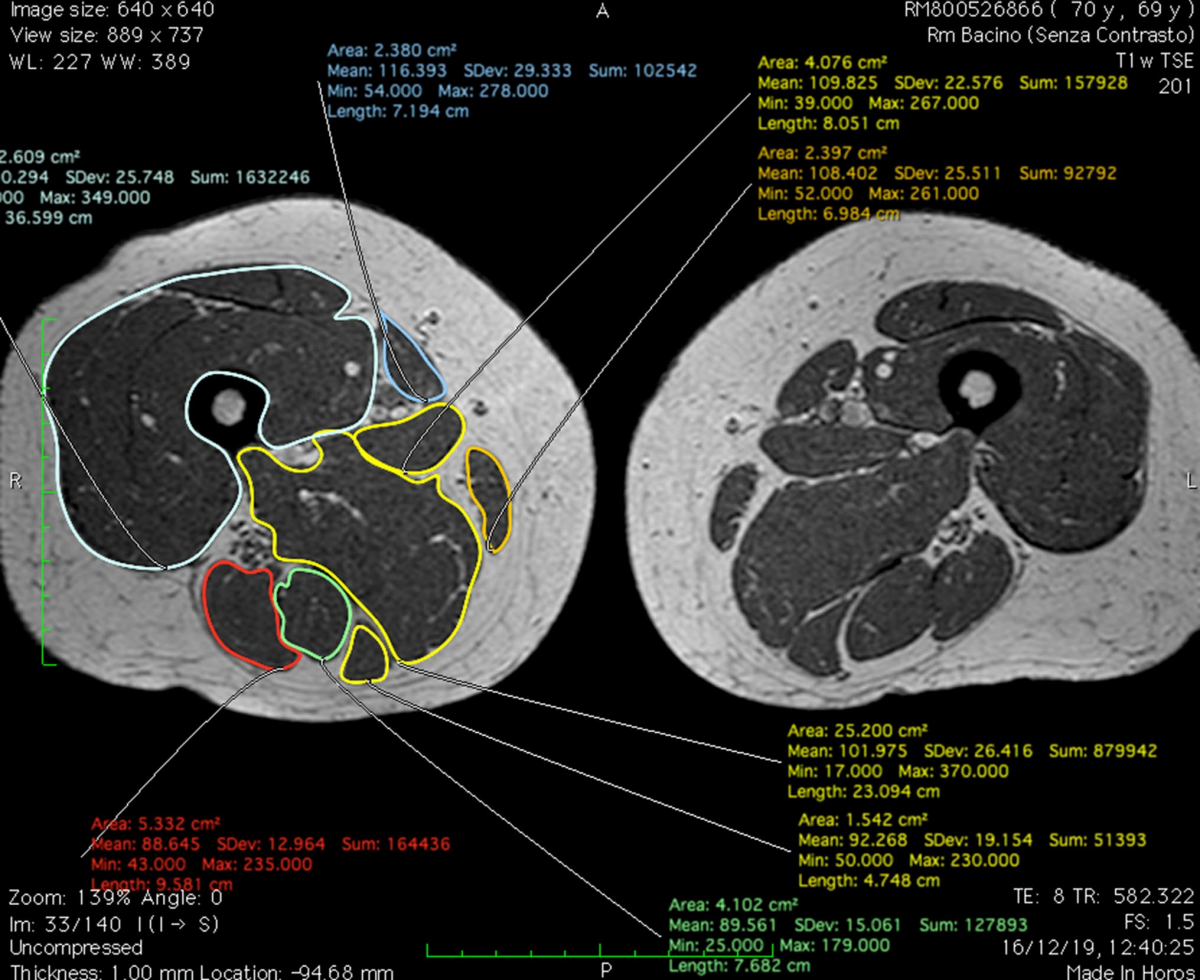
Representative assessment of cross-sectional area on magnetic resonance image of the thigh with rectus femoris, vastus lateralis, vastus intermedius and vastus medialis labeled, using “closed polygon” function (software Horos)

MRI is the gold standard for muscle assessment [88–90]. In addition, MRI is non-invasive and extremely reliable technique. MRI allows high-contrast distinction of soft tissue components (muscle, fat mass, and water) depending on anatomical compartment molecular characteristics. MRI may identify changes in muscle composition, such as muscle disruption, edema, or intramuscular adipose tissue (myosteatosis) and fibrosis (myofibrosis), as well as other biochemical indicators related to muscle quality. Intramuscular adipose tissue is made up of intermuscular and intramuscular fat. Anatomical T1- and T2-weighted sequences are often employed to assess muscle fat content. Anatomical imaging can measure CSA and muscle volume. These measures may identify hypertrophy or atrophy [91, 92]. Semiquantitative MRI muscle atrophy and fat infiltration methods have been published. The semiquantitative CT ratings may also apply to MRI (Fig. 3). Based on the amount of intramuscular fat visible on CT scans, Goutallier and colleagues were the first to describe fatty infiltration grades of the shoulder rotator cuff muscles on a five-point scale (0 = normal muscle; 1 = the muscle contains some fatty streaks; 2 = the fatty infiltration is important, but there is still more muscle than fat; 3 = there is as much fat as muscle; 4 = more fat than muscle is present) [93]. Modifications of the Goutallier classification to evaluate muscle volume and fat infiltration have recently been suggested for MRI with better reliability [94–98] (Fig. 4).

MRI can assess muscle amount as well as quality. Advanced MRI methods, unlike standard T1- and T2-weighted sequences, enable quantification of muscle composition and imaging of sarcopenia biomarkers [68]. By separating signal in each voxel of tissue, Dixon sequences enable for precise measurements of muscle volume and fat infiltration [99, 100]. Water-fat MRI may detect intermuscular and intramuscular fat separately. Automatic segmentation for determining whole body and regional muscle volume was suggested using Dixon MRI sequences [101]. The Dixon method is a chemical shift-based MRI sequence created to achieve homogeneous fat suppression. It consists in the acquisition of in-phase and out-of-phase images from which water-only and fat-only images are reconstructed, allowing for precise measurements of muscle volume and the degree of fat infiltration. It has the added advantage of providing both fat-suppressed and non-fat-suppressed images in a single acquisition, and it has been used in association with fluid-sensitive sequences.

MRI spectroscopy may also be used to properly assess intracellular lipid levels, which might be elevated in diseases like cancer or insulin resistance [66, 102]. MRI spectroscopy employs H1 proton signals to examine molecular tissue components. 1H MRI spectroscopy is a magnetic resonance-based chemical analytical technique which offers the possibility to specifically quantify the percentage of intracellular fat in a certain volume of interest (VOI). It is used in in organic chemistry to identify structural compounds and has the advantage of giving additional metabolic information, but it is associated with considerable sample error as a consequence of VOI position variance, because small changes in the VOI position may have a great impact on accurate fat quantification. However, is still often considered as the gold standard of volumetric fat quantification. Distinctive MR approaches for measuring subcutaneous and visceral fat, as well as fat inclusion in various tissues and organs [103, 104]. In the past, these methods were used to measure liver fat and other diseases like muscular dystrophy [105, 106]. For measuring muscle fat in sarcopenic males, Grimm and colleagues used Dixon MRI and multi-echo magnetic resonance spectroscopy [107]. The Dixon sequencing and spectroscopy indicated good correlations and accuracy for thigh fat measurements. Dixon sequences map fat distribution while spectroscopic measures localized fat. However, since MR whole-body imaging with morphological and functional imaging acquisition processes is time-consuming and expensive, it may not be practicable in most clinical settings. As a consequence, numerous anatomical, representational levels or muscles have been found as alternatives to whole-body techniques [101, 108–111]. Schweitzer and coworkers claim the third lumbar vertebra level has the most repeatability and bodily compartment connection [112]. Thigh muscles are a good candidate for MRI research because of their great magnetic field homogeneity, minimal motion artifacts, and association with physical function in older people.

**Fig. 5:**
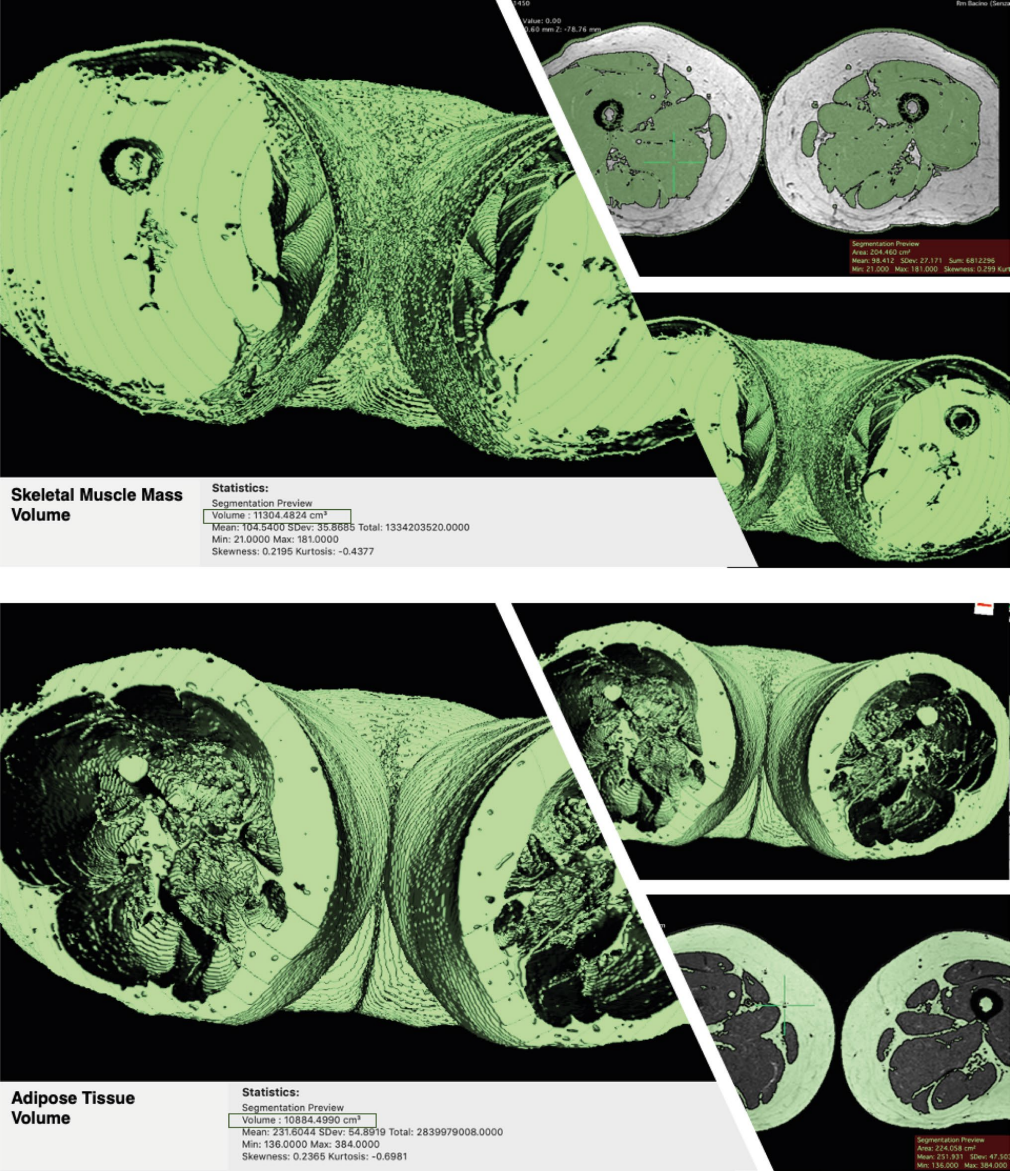
Skeletal muscle mass and adipose tissue volume reconstruction (software Horos) on MRI images. The quadriceps muscle volume reconstruction is based on the selection of the T2w dataset, selection of the command to generate the “Region of Interest,” insertion of the threshold interval, selection of the parameter “3D Growing region,” so that all the slices are considered, and application of the command “Brush ROI” to obtain a complete segmentation

An MRI sequence including a region of interest (ROI) may be used to segment muscles and fat to quantify muscle and fat volume. Another study recommended measuring muscle volume from a single leg section to save money and effort. Selected anatomical landmarks' CSA have been found to be good surrogates for total skeletal muscle amount [113]. These findings are consistent with those of Yang and colleagues [114]. The authors predicted that a single MRI segment may represent the whole thigh. Other studies have linked CSA to quadriceps, hamstrings, and adductors [115, 116]. Because CSA may be recorded in a single slice rather than segmenting the whole thigh muscle volume, this can drastically reduce costs, scan time, and post-processing time. Semiquantitative evaluations have been proven to be less reliable than CSA measurements and advanced quantitative segmentation algorithms in 2D or 3D [117, 118]. Manual muscle segmentation is repeatable yet time demanding, limiting its utility in large-scale research. Muscle and other tissues have recently been quantified utilizing semiautomatic and automated threshold changes in pixels/voxels [119, 120]. Our research uses the open-source program Horos (version 3.3.6 for macOS 10.11 +), which is based on OsiriX and other open-source technologies, to semiautomatically segment the quadriceps muscle CSA and pixel-based intensity (PI). Dedicated software like Horos allows 3D segmentation with QMV reconstruction and differentiation of skeletal muscle mass and adipose tissue volume (Fig. 5).

The use of artificial intelligence-based quantitative image analysis for muscle mass segmentation may improve the current standard of care. Artificial intelligence-based quantitative image analysis, which includes machine learning and deep learning, has been proposed for automated and accurate abdominal fat tissue evaluation. It is a set of methods that allow computers to learn from data and extrapolate or categorize models. Machines may be able to analyze enormous volumes of data and extract characteristics that humans cannot. An artificial neural network (ANN) approach uses a multilayered structure to obtain high-level abstractions in data. These data are more and more useful for clinicians treating a broad range of illnesses, including cardiovascular and oncologic disorders, to evaluate risk, etiology, clinical outcomes, treatment response, and complications [121–123].

## Conclusion

Rheumatologists and radiologists have a pivotal role in sarcopenia diagnosis [124–126]. Medical examination for sarcopenia diagnosis has certain disadvantages. BIA may be affected by age, gender, hydration status, and ethnicity [127]. Comorbidity, musculoskeletal issues, and cognitive impairments may make functional assessments and grip strength difficult [128]. Age-related variables, such as the loss of degenerative disk thickness and the associated height decrease, might affect BMI. Using a combination of US and MRI to check the lower leg muscles might be a simple and painless technique to diagnose sarcopenia in patients with IMRDs. Although the application potential is appealing, further study is required to establish a robust evidence foundation and a consistent approach. To prove its validity and reliability, it must be thoroughly compared to other gold-standard data, as well as a normative data collection for the creation of low muscle mass measures. Because it does not need the removal of clothing, forearm muscle depth may be a particularly valuable diagnostic approach [129]. US might be a valuable tool in a physician's toolbox, allowing for more accurate sarcopenia diagnosis and more effective diet and exercise treatment [126, 130]. Aside from the apparent benefits for the patient and caregiver, health care costs are minimal, and savings are significant. The potential advantages of muscle screening using US and MRI for older people should be investigated further.
